# Integrating Wearable Sensor Signal Processing with Unsupervised Learning Methods for Tremor Classification in Parkinson’s Disease

**DOI:** 10.3390/bioengineering12010037

**Published:** 2025-01-06

**Authors:** Serena Dattola, Augusto Ielo, Angelo Quartarone, Maria Cristina De Cola

**Affiliations:** IRCCS Centro Neurolesi Bonino-Pulejo, S.S. 113 Via Palermo, C. da Casazza, 98124 Messina, Italy; serena.dattola@irccsme.it (S.D.); angelo.quartarone@irccsme.it (A.Q.); mariacristina.decola@irccsme.it (M.C.D.C.)

**Keywords:** unsupervised learning, tremor detection, Parkinson’s disease, wearable sensors

## Abstract

Tremor is one of the most common symptoms of Parkinson’s disease (PD), assessed using clinician-assigned clinical scales, which can be subjective and prone to variability. This study evaluates the potential of unsupervised learning for the classification and assessment of tremor severity from wearable sensor data. We analyzed 25 resting tremor signals from 24 participants (13 PD patients and 11 controls), focusing on motion intensities derived from accelerometer recordings. The k-means clustering algorithm was employed, achieving a classification accuracy of 76% for tremor versus non-tremor states. However, performance decreased for multiclass tremor severity classification (57.1%) and binary classification of severe versus mild tremor (71.4%), highlighting challenges in detecting subtle intensity variations. The findings underscore the utility of unsupervised learning in enabling scalable, objective tremor analysis. Integration of such models into wearable systems could improve continuous monitoring, enhance rehabilitation strategies, and support standardized clinical assessments. Future work should explore advanced algorithms, enriched feature sets, and larger datasets to improve robustness and generalizability.

## 1. Introduction

Parkinson’s disease (PD) is a neurodegenerative disease of the central nervous system. Symptoms begin slowly. The first symptom may be a barely perceptible tremor in one hand, or sometimes in one foot or jaw. Although PD presents a wide range of symptoms, the most common are motor-related, including tremor, slowness of movement, rigidity, and difficulty with balance [1]. Several non-motor manifestations including dementia and mood alteration which affect quality the of life of patients and their caregivers may also arise in advanced stages [2,3].

Parkinson’s disease symptoms can be different for everyone. Early symptoms may be mild and unrecognized. They also often start on one side of the body and then affect both sides, but usually one side is more affected than the other. Typical resting tremor is the most common symptom [4]. It therefore plays a key role in the diagnosis of PD, which is based on the patient’s medical history and a neurological and physical examination where symptoms are investigated [5]. The most common clinical scales used to follow the longitudinal course of PD are the Hoehn and Yahr scale [6] and the Movement Disorder Society’s unified Parkinson disease rating scale (MDS-UPDRS) [7]. However, the assessment involves scoring based on the severity of the disease by interview and clinical observation. Therefore, the evaluation may be subjective and affected by variability, with reported misdiagnosis rates up to 37% [8] or even 50% [9]. This uncertainty reflects the need for more objective measures for tremor classification.

In the last decade, research into human movement monitoring has mainly focused on the development of increasingly accurate and non-invasive sensor devices to collect data at a low cost [10], and increasingly sophisticated machine learning (ML) algorithms to process these data using different types of features extracted from sensor signals [11,12]. In the context of ML, measurement devices provide objective measures of tremor movement from which features can be extracted as quantifiable characteristics and used as input to an algorithm. Therefore, ML methods, which ‘learn’ patterns from data without human instruction, can provide a great benefit to tremor research.

The scientific literature contains several techniques to quantify and characterize tremor, and accelerometry is by far the most widely used method [13], although only a few link accelerometer data to a corresponding clinical score. For instance, the MDS-UPDRS includes several sections assessing the effects of the disease in different aspects and its score of 3.17 indicates the amplitude of tremor at rest. In addition, they often perform supervised ML, exploiting the disease severity score assigned by a neurologist (e.g., the MDS-UPDRS score) to label the instances used to train the model [12,14,15,16], with the risk of using biased data due to individual physician scoring affecting the performance of the ML models [17]. In contrast, unsupervised machine learning techniques have the advantage of learning from accelerometer data to identify meaningful patterns of different types of tremor severity. This avoids the need for the provision of training data labeled with tremor severity, which can be both time-consuming and expensive to create [18].

Clustering techniques are widely employed in unsupervised learning, especially within the field of human activity recognition [19,20]. Among these techniques, k-means clustering stands out as a prevalent partitioning method used to uncover natural groupings within data. While k-means is renowned for its simplicity and computational efficiency, its performance is sensitive to the initial placement of centroids and can be affected by outliers or clusters with varying shapes and sizes [21]. Despite these limitations, k-means remains a well-established, computationally efficient, and widely used method in exploratory data analysis and pattern recognition, providing valuable insights into the underlying structure of data [22,23,24,25].

This study explores the use of an unsupervised learning model, k-means, to face two classification problems: (i) the distinction of patients from controls, i.e., distinguishing between tremor and non-tremor using a k = 2 cluster; and (ii) the distinction of different tremor severities, where we classified the levels of tremor severity in PD patients using a multiclass classification with k = n. Finally, we also performed a third classification problem with the aim of distinguishing between severe tremor vs. mild tremor (i.e., k = 2), in order to simplify the second problem.

## 2. Materials and Methods

In this section, we describe the dataset we used and all the steps of our analysis, including the ML process that is based on k-means clustering.

### 2.1. Dataset Description

The dataset used in this study is publicly accessible through IEEE DataPort at https://doi.org/10.21227/g2g8-1503 (accessed on 29 April 2024) [26]. Data were collected using lightweight MC10 BioStamp RC sensors (MC10 Inc., Lexington, MA, USA) to measure activity, gait, and tremor in individuals with PD in both clinical settings and real-world environments. The dataset comprises recordings from 17 individuals diagnosed with PD (mean age ± standard deviation: 66.4 ± 11.3 years; 41.2% female) and 17 age-matched healthy control subjects (HCs = 64.0 ± 9.9 years; 76.5% female). Participants were equipped with five adhesive sensors, one affixed to each limb and the trunk, capturing triaxial accelerometer data at a sampling frequency of 31.25 Hz. Data collection occurred during a clinic visit (supplemented by annotation files) and extended over two additional days in the participants’ daily environment. In addition, subjects underwent a standard clinical evaluation using the Movement Disorder Society’s Unified Parkinson’s Disease Rating Scale (MDS-UPDRS) Part III [7], the Timed Up-and-Go test [27], and the Ten-Meter Walk test [28].

As our aim was to classify tremor, we used the MDS-UPDRS 3.17 scores, which assess the amplitude of tremor at rest. In the 3.17 MDS-UPDRS item, patients sit quietly in a chair for 10 s with their hands resting on the chair arms and feet flat on the floor. The physician assesses resting tremor separately in all four limbs and the lips/jaw, assigning a score between 0 and 4, as reported in Table 1. The final score corresponds to the maximum tremor amplitude observed. For HCs, clinicians assess the right hand and record a maximum at-rest tremor score of 0 on the MDS-UPDRS.

In this study, we considered only the scores pertaining to the upper limbs of PD subjects. Therefore, we excluded 4 PD patients due to missing MDS-UPDRS 3.17 scores, and 6 HCs because their annotation files lacked the necessary information for our analysis (specifically, the start and end timestamps of clinical assessments in a resting state). Thus, our population included 13 PD patients (mean age ± SD: 66.1 ± 11.8 years; 38.5% female) and 11 HCs (66.0 ± 8.4 years; 90.1% female). For each PD patient, we identified and extracted recordings from the tremor-predominant arm. For one PD patient, we found that both arms had a tremor of equal severity (i.e., with the same score), and we decided to include both of these recordings. As a result, the final dataset included 14 recordings from the PD patients and 11 recordings from the HCs, for a total of 25 recording instances.

### 2.2. Data Pre-Processing

Feature pre-processing is an essential step for most machine learning algorithms. Pre-processing usually involves transforming the input data to a form that is more suitable for the learning algorithm, in order to improve its performance.

At first, we performed a data segmentation process with the aim of extracting time intervals in which subjects were seated in a resting state. As mentioned above, an annotation file was provided for each participant, detailing the start and end timestamps of the tasks performed during the clinical assessment. We used these annotations to extract the resting periods from the time series. The extracted segments were concatenated into a single string, i.e., a recording instance. No additional resting periods were reported on the two-day records, so these days were not included in the analysis.

Next, to transform the data into a suitable form for the k-means unsupervised clustering algorithm, we adjusted for the effects of sensor orientation and individual bias. Therefore, we performed two additional pre-processing procedures, the well-known mean-centering and the modulus calculation.

#### 2.2.1. Mean-Centering

From a geometric point of view, data centering is just a translation or repositioning of the coordinate system. In other words, the mean-centering procedure corresponds to moving the origin of the coordinate system to coincide with the average point.

Let $a^{i}=\left\{\left(x_{j},y_{j},z_{j}\right):j=1,\ldots ,n_{i}\right\}$ be the raw accelerometer data of the recording instance *i*, which contains $n_{i}$ samples, according to the length of the resting period in *i*. Then, we centered each data point $a^{i*}=\left\{\left(x_{j}^{*},y_{j}^{*},z_{j}^{*}\right):j=1,\ldots ,n_{i}\right\}$ in *i* by subtracting the average value ${\bar{a}}^{i}=\left(\bar{x},\bar{y},\bar{z}\right)$ calculated as

$${\bar{a}}^{i}=\left(\frac{\sum_{j=1}^{n_{i}}x_{j}}{n_{i}},\frac{\sum_{j=1}^{n_{i}}y_{j}}{n_{i}},\frac{\sum_{j=1}^{n_{i}}z_{j}}{n_{i}}\right)$$

from each sample *j* as follows:

$$a_{j}^{i*}=\left(x_{j}-\bar{x},\;y_{j}-\bar{y},\;z_{j}-\bar{z}\right)\;\forall \;j=1,\ldots ,n_{i}$$

where $n_{i}$ is the number of samples in the recording instance *i*.

After mean-centering, each element of the mean-centered data $a^{i*}$ is derived from the continuous component, allowing us to remove constant distortions and emphasize dynamic movements, which are more relevant for tremor detection.

#### 2.2.2. Modulus of Mean-Centered Data

Subsequently, to capture the overall intensity of movement, regardless of direction, we calculated the modulus of all mean-centered accelerometer data $∥a^{i*}∥$, for each sample *j* as follows:

$$∥a_{j}^{i*}∥=\sqrt{{x_{j}^{*}}^{2}+{y_{j}^{*}}^{2}+{z_{j}^{*}}^{2}}\;\forall \;j=1,\ldots ,n_{i}$$

where $n_{i}$ is the number of samples in the recording instance *i*.

In this way, we had a scalar representation of the movement intensity, simplifying the data and preserving the essential information of magnitude.

### 2.3. K-Means Clustering Model

The data were concatenated into a single dataset containing all $n_{i}$ moduli $∥a_{j}^{i*}∥$ for each recording instance *i*. A k-means clustering was then applied.

The k-means is a popular clustering algorithm that partitions a dataset into *k* distinct and non-overlapping clusters. The centers of the initial *k* cluster (centroids) are usually either initialized randomly or, when the first centroid is initialized randomly, then the other centroids are chosen in such a way as to be spread out as much as possible, i.e., through heuristic methods (known as K++ [21]). Next, k-means associates each data point $a_{j}^{i*}$ (i.e., its modulus) with the nearest centroid $\mu_{w}$ based on a chosen distance metric, forming clusters accordingly. We chose the Euclidean distance as the distance metric because of its effectiveness in measuring the similarity of the movement intensities represented by the modulus values [29]. The set of points belonging to cluster *u* is shown as $C_{u}$:

$$C_{u}=\{∥a_{j}^{i*}∥:a_{j}^{i*}-\mu_{u}\leq ∥a_{j}^{i*}-\mu_{w}∥\}$$

The centroids are recalculated iteratively as the mean of the points within each cluster:

$$\mu_{u}=\frac{1}{|C_{u}|}$$

where $|C_{u}|$ is the number of points in the cluster *u*, and the assignment step is repeated until convergence is achieved, effectively minimizing the within-cluster variance [30]. Thus, the algorithm’s second step recalculates the centroids of each cluster to minimize the sum of squared Euclidean distances from the data points of the cluster to the cluster centroid.

#### Percentile Reduction

As tremor events may not be evenly distributed across subjects, we extracted only the periods with the highest movement intensities. Specifically, for each recording instance *i*, we calculated percentiles in the distribution of moduli $∥a^{i*}∥$ and selected those with values above p=0.95 (i.e., the 95th percentile). In this way, we assumed that tremor episodes would correspond to peaks in movement intensity, and that focusing on these peaks would enhance the sensitivity of the clustering algorithm to detect tremor-related patterns. We then determined the ’dominant cluster’ by identifying the cluster label that occurred most frequently in the top 5% of modulus instances.

### 2.4. Classification Performance Evaluation

After clustering, we evaluated the performance of the k-means algorithm by comparing the cluster assignments to the actual tremor labels (for the binary classification) or tremor severity scores (for the multiclass classification) according to the score of the 3.17 MDS-UPDRS item. No patients graded 4 on clinical assessment were reported.

**Figure 1:**
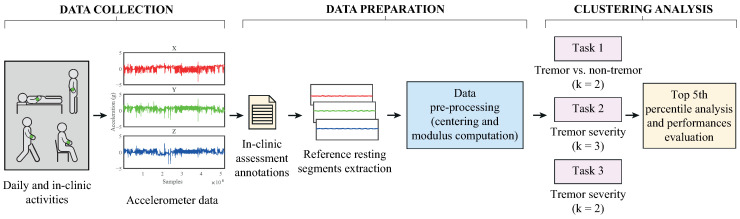
Flow diagram of the study.

Therefore, our idea was to use the clinical information provided by the 3.17 MDS-UPDRS task (i.e., resting tremor) as a label for each recording instance, and to compare it with the one assigned by the classifier. However, k-means clustering is unsupervised and does not produce labeled clusters. Thus, we used a permutation mapping (best matching) approach to align the cluster labels with the true labels. Permutation mapping involves finding the best one-to-one mapping between the cluster labels and the true labels that maximizes the overall accuracy. We tested all possible permutations of cluster label assignments and selected the mapping that resulted in the highest accuracy percentage. The data preparation process and clustering analysis are described in Figure 1.

Evaluating the performance of a classification model is critical to ensuring its efficiency. One of the most common statistical measures of classification performance is the accuracy, which is the proportion of correct predictions (both true positives and true negatives) among the total number of instances. Therefore, we evaluated model performance in binary classification as

$$Accuracy=\frac{TP+TN}{TP+TN+FP+FN}$$

where *TP* = true positive; *FP* = false positive; *TN* = true negative; *FN* = false negative. Such values have been determined by comparing the predicted labels (i.e., the assigned clusters) against the true labels (the 3.17 MDS-UPDRS scores) in each class and recording instance.

In multiclass classification, accuracy is defined as

$$Accuracy=\frac{\mathrm{correctly}\;\mathrm{classified}\;\mathrm{instances}}{\mathrm{total}\;\mathrm{number}\;\mathrm{of}\;\mathrm{instances}}$$

The performance of classification tasks was further evaluated using the following key metrics: precision, recall, and F1 score.

Precision is defined as the proportion of true positive predictions out of all instances that were predicted as positive. Mathematically, precision is expressed as

$$Precision=\frac{TP}{TP+FP}$$

High precision indicates that the model rarely misclassifies negative instances as positive. This metric is particularly important in contexts where false positives carry a significant cost, such as in medical diagnoses or fraud detection.

Recall measures the proportion of true positive instances that were correctly identified by the model. The formula for recall is

$$Recall=\frac{TP}{TP+FN}$$

High recall indicates that the model is effective at identifying positive instances, even if it occasionally misclassifies negatives as positives. This metric is particularly valuable in situations where missing positive cases (false negatives) is costly, such as in disease screening.

The F1 score is the harmonic mean of precision and recall, combining these two metrics into a single value to provide a balanced assessment of the model’s performance. It is calculated as

$$F1=2\cdot \frac{Precision\cdot Recall}{Precision+Recall}$$

The F1 score is particularly useful when there is an imbalance between the positive and negative classes, as it ensures that both precision and recall are considered equally important. A high F1 score indicates that the model performs well in balancing the trade-off between precision and recall.

These metrics provide a comprehensive understanding of the strengths and weaknesses of the classification model by assessing its ability to correctly identify positive instances and minimize false predictions. For the multiclass task, precision, recall, and F1 score were computed for each score and then averaged.

By employing these metrics, we aim to provide a detailed evaluation of the classification models used in this study, identifying their ability to accurately classify instances while minimizing errors. Furthermore, confusion matrices were used to visually describe the performance of any classification model.

All analyses in this study were conducted using MATLAB R2024a (MathWorks, Natick, MA, USA).

## 3. Results

We performed two classification problems: (1) binary classification distinguishing tremor from non-tremor states, and (2) multiclass classification of tremor severity levels among PD patients. In addition, we simplify the second problem by performing a binary classification comparing higher tremor (score 3) against milder tremor scores (scores 1 and 2 combined). For the sake of clarity, we refer to Task 1 as the first problem, Task 2 as the second problem, and Task 3 as binarizing the latter.

### 3.1. Tremor vs. Non-Tremor Classification

The algorithm achieved an accuracy of 76.0%, correctly classifying 19 out of 25 recording instances. Specifically, most PD patients were correctly assigned to the tremor cluster, while the majority of HCs were assigned to the non-tremor cluster. However, five PD patients were misclassified into the non-tremor cluster, indicating limitations in the clustering approach for certain individuals. The model achieved a precision of 1.00 and a recall of 0.57, resulting in an F1 score of 0.73.

Table 2 presents the clustering results for each subject.

### 3.2. Tremor Severity Classification

#### 3.2.1. Multiclass Classification

The algorithm achieved an accuracy of 57.1%, correctly classifying 8 out of 14 instances. The performance varied across classes: Score 1 had a precision of 0.67, recall of 0.67, and F1 score of 0.67; Score 2 achieved a precision of 0.50, recall of 0.60, and F1 score of 0.55l; and Score 3 showed a precision of 0.50 and a recall of 0.33, leading to an F1 score of 0.40. The average precision, recall, and F1 score were 0.56, 0.53, and 0.54, respectively. The clustering algorithm struggled to accurately distinguish between the different tremor severity levels, with significant overlap in cluster assignments. Looking at the results, it is noticeable that subjects with 3.17 MSD-UPDRS tremor scores of 1 and 2 were often grouped together in the same cluster, while subjects with higher tremor scores were often clustered with those with lower scores, as shown in Table 3.

#### 3.2.2. Binary Classification of Higher vs. Milder Tremor Scores

The algorithm achieved an accuracy of 71.4%, correctly classifying 10 out of 14 instances, with a precision of 0.40, recall of 0.67, and F1 score of 0.50. The results show an improvement over the multiclass classification, as viewable in Table 4.

**Figure 2:**
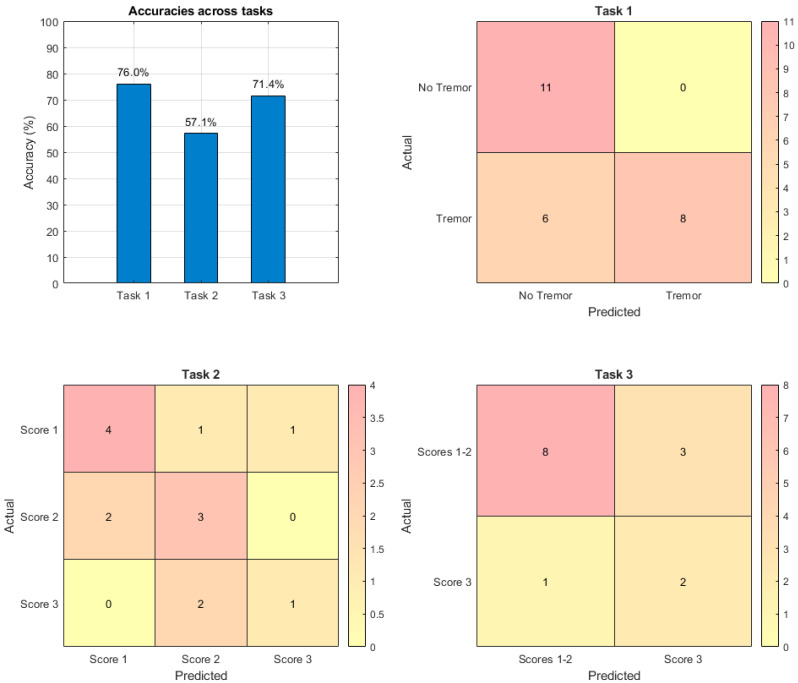
Classification task performances. The **top left** panel displays a bar chart summarizing the accuracy of each classification task: tremor vs. non-tremor (76.0%), multiclass tremor severity (57.1%), and severe vs. milder tremor (71.4%). The remaining panels provide confusion matrices for each classification task, illustrating the distribution of predictions. The **top right** panel shows the binary classification of tremor vs. non-tremor, the **bottom left** shows the multiclass classification of different tremor severity levels, and the **bottom right** shows the binary classification of lower versus higher tremor severity.

Table 5 summarizes the performance metrics of the classification algorithms, while Figure 2 shows the accuracies and confusion matrices across different tasks.

## 4. Discussion

Tremor is the most prevalent symptom of Parkinson’s disease and initially tends to affect only one side of the body. Resting tremor typically occurs when the limbs are not actively engaged in movement, such as when the patient is seated or walking with arms hanging loose. In this study, we exploited the Rest Tremor Amplitude item (MDS-UPDRS 3.17) to analyze data from 24 participants (13 with PD and 11 controls). The signals, recorded by wearable devices, were obtained during standard diagnostic exercises conducted by neurologists.

Wearable sensors are the most frequently used devices to detect and assess tremors in Parkinson’s disease. However, they are not the only technology used for this purpose. Advanced methods such as computer vision algorithms [31] and deep learning applied to video analysis [32] enable precise tremor detection without physical sensors. Additionally, optical motion capture systems with infrared cameras allow for detailed motion tracking [33].

The scientific literature extensively documents tremor analysis in PD using supervised ML approaches applied to accelerometer data extracted from sensors (although unsupervised ML, such as dimensionality reduction, is sometimes used as a precursor step) [13,34,35,36]. However, labels derived from clinical assessments are subjective and may vary between clinicians due to differences in training, experience, and interpretation of patient symptoms. This variability introduces noise and potential bias into the dataset, which can negatively affect the performance and generalizability of supervised machine learning models [17]. Unlabeled data, on the other hand, allow algorithms to identify patterns and groupings directly from the intrinsic structure of the data without relying on external annotations. This approach ensures that results are driven by the raw, objective characteristics of the input features, rather than potentially inconsistent or biased labels. Thus, unsupervised learning can provide additional insights into tremor data [37]. This perspective lead us to investigate on the potential of unsupervised learning by using the k-means clustering algorithm to classify tremor severity using accelerometer data from PD patients and health controls.

To our knowledge, this is the first study that applies an unsupervised learning model to analyze resting tremor severity in PD patients. Our findings show that the k-means clustering algorithm successfully classified tremor versus non-tremor states with an accuracy of 76.0%, demonstrating its efficacy in capturing fundamental differences in motion patterns. However, its performance was lower in multiclass classification, reaching about 57.1% accuracy. But when we made the classification problem binary (i.e., moderate versus mild tremor), we found that the performance of the method increased until reaching about 71.4% accuracy, suggesting challenges in distinguishing subtle variations in tremor intensity. The classification performance highlights the capability of k-means clustering to process unlabeled motion data. Furthermore, focusing on the top 5% of movement intensities improved sensitivity to tremor patterns, underscoring the importance of effective feature extraction in machine learning applications.

The ability to classify using unsupervised learning has important implications for clinical practice [38,39]. Similarly, methods concerning the classification of tremor states and severity in PD could supplement traditional clinical evaluations, providing a more objective and reproducible measure of motor symptoms. For instance, integrating clustering-based tools into wearable devices could enable continuous monitoring of PD symptoms, supporting early intervention and treatment adjustments. Moreover, unsupervised methods could address challenges associated with variability in clinician-assigned scores. By directly analyzing raw motion data, these algorithms reduce the risk of subjective bias, paving the way for standardized assessments across different clinical settings. Beyond the objectivity of an unsupervised learning approach, the main key strength of this study lies in the simplicity of the model used. The simple pre-processing steps (mean-centering and modulus calculations) effectively normalized individual biases and amplified dynamic movements, contributing to the model’s accuracy. In addition, the percentile analysis approach included in the clustering model has reduced the impact of non-tremor movements and random noise on classification performance, as the highest modulus values correspond to motor activity associated with tremor. On the other hand, this study has some limitations. First of all, the analysis was performed on a relatively small dataset (25 recording instances). This limited sample size may have restricted the generalizability of the results, especially for multiclass classification tasks. Moreover, while the focus on the top 5% of motion intensities improved sensitivity, the exclusion of temporal- and frequency-domain features limited the algorithm’s ability to capture subtle differences in tremor dynamics. Finally, the k-means algorithm’s performance is sensitive to initialization and assumes spherical cluster shapes, which may not align with the true distribution of tremor data. However, it is worth noting that our study employed one-dimensional data derived from the modulus calculation, which simplified the clustering problem and made it more manageable. Repeating the study on a larger dataset might be essential to confirm our results.

The findings of this study could also have implications for the field of rehabilitation in PD [40,41]. Real-time analysis of tremor severity using wearable devices could provide immediate feedback to both patients and therapists during physical therapy sessions, allowing for adjustments of the exercises based on the patient’s motor abilities and needs. This dynamic feedback could enhance the efficacy of rehabilitation programs through targeted activities that align with the patient’s tremor patterns. Furthermore, continuous monitoring of tremor severity in home-based settings could help clinicians track the effectiveness of rehabilitation interventions over time. By exploiting unsupervised models, rehabilitation programs could also be adapted based on objective data rather than relying solely on patient self-reports or sporadic clinical assessments, which are often subject to bias and variability. This approach could support the development of remote, technology-assisted rehabilitation protocols, allowing for more consistent management of PD symptoms and potentially improving patients’ quality of life.

The misclassification resulting from the evaluated performance metrics highlights the presence of individual differences in tremor patterns that were not fully captured by the current model. These differences likely arise from variations in patient-specific motor symptoms, disease progression, or recording conditions, which can introduce noise and variability into the data. In practical applications, such misclassifications could lead to an inaccurate assessment of a patient’s condition. To address the limitations and enhance the applicability of the findings, future research could investigate several promising directions. Advanced clustering algorithms, such as hierarchical clustering, may be more effective in handling irregular cluster shapes and overlapping boundaries [42]. Hybrid models that combine unsupervised clustering with supervised learning, such as semi-supervised approaches, could utilize small amounts of labeled data to improve cluster quality while maintaining scalability [43]. Enriching features by incorporating temporal, frequency-domain, and multimodal data, including neuroimaging and biomarkers, could increase the algorithm’s sensitivity to subtle differences in tremor severity. Finally, expanding the dataset to include recordings from a more diverse cohort of PD patients and healthy controls would improve the generalizability and robustness of the findings. Incorporating longitudinal data could further demonstrate the algorithm’s utility in tracking disease progression over time.

## 5. Conclusions

Machine learning is increasingly playing a significant role in clinical assessments of neurodegenerative diseases. In particular, this study highlights the feasibility of using unsupervised learning methods, such as k-means clustering, for the classification of tremor states and severity in PD using wearable sensor data. The methodology introduced in this research emphasizes simplicity and adaptability, making it suitable for integration into wearable systems for continuous symptom monitoring. The approach successfully demonstrated that raw motion data can provide valuable insights without relying on predefined labels, achieving good accuracy in distinguishing tremor from non-tremor states. However, the results for tremor severity classification, especially in multiclass scenarios, show the complexities of subtle tremor differentiation. Therefore, while the findings suggest the potential utility of unsupervised models in improving traditional clinical evaluations, they also point to the importance of enhancing feature extraction and algorithm design for greater accuracy. The application of unsupervised learning to PD tremor analysis represents a step toward more data-driven, patient-centered care. As sensor technologies continue to evolve, the integration of real-time data analytics into clinical workflows could improve patient treatment. These results open the way for future research to refine the algorithms and explore broader applications in neurodegenerative disease management. By focusing on scalability and precision, this approach could change the assessment and management of motor symptoms, contributing to better patient outcomes.

## Figures and Tables

**Table 1:** UPDRS 3.17 extremity severity scoring.

Severity	Description	Score
Normal	No tremor	0
Slight	<1 cm in maximal amplitude	1
Mild	≥1 cm but <3 cm in maximal amplitude	2
Moderate	≥3 cm but <10 cm in maximal amplitude	3
Severe	≥10 cm in maximal amplitude	4

**Table 2:** Clustering results for binary classification of tremor vs. non-tremor.

Instance ID	MDS-UPDRS Score	Predominant Cluster	Correctly Classified
1	1	1	Yes
2	1	1	Yes
3	1	1	Yes
4	1	1	Yes
5	1	0	No
6	1	1	Yes
7	1	0	No
8	1	0	No
9	1	0	No
10	1	0	No
11	1	1	Yes
12	1	1	Yes
13	1	1	Yes
14	1	0	No
15	0	0	Yes
16	0	0	Yes
17	0	0	Yes
18	0	0	Yes
19	0	0	Yes
20	0	0	Yes
21	0	0	Yes
22	0	0	Yes
23	0	0	Yes
24	0	0	Yes
25	0	0	Yes

Legend score: tremor = 1; non-tremor = 0.

**Table 3:** Clustering results for tremor severity classification among PD patients.

Instance ID	MDS-UPDRS Score	Predominant Cluster	Mapped Score	Correctly Classified
1	2	3	2	Yes
2	3	3	2	No
3	2	3	2	Yes
4	2	3	2	Yes
5	1	1	1	Yes
6	3	2	3	Yes
7	2	1	1	No
8	1	1	1	Yes
9	1	1	1	Yes
10	2	1	1	No
11	3	3	2	No
12	1	3	2	No
13	1	2	3	No
14	1	1	1	Yes

Legend severity score: 1 = slight; 2 = mild; 3 = moderate.

**Table 4:** Clustering results for binary tremor severity classification among PD patients.

Instance ID	MDS-UPDRS Binary Score	Predominant Cluster	Correctly Classified
1	1	2	No
2	2	1	No
3	1	1	Yes
4	1	1	Yes
5	1	1	Yes
6	2	2	Yes
7	1	1	Yes
8	1	1	Yes
9	1	1	Yes
10	1	1	Yes
11	2	2	Yes
12	1	2	No
13	1	2	No
14	1	1	Yes

Legend severity score: 1 = slight or mild; 2 = moderate.

**Table 5:** Performance evaluation across different tasks.

	Accuracy	Precision	Recall	F1 Score
Task 1	0.76	1.00	0.57	0.73
Task 2	0.57	0.56	0.53	0.54
Task 3	0.71	0.40	0.67	0.50

## Data Availability

The original data presented in the study are openly available in PD-BioStampRC21 at https://doi.org/10.21227/g2g8-1503 (accessed on 29 April 2024).
